# Ultrafast transverse relaxation exchange NMR spectroscopy†

**DOI:** 10.1039/d2cp02944h

**Published:** 2022-09-02

**Authors:** Md Sharif Ullah, Otto Mankinen, Vladimir V. Zhivonitko, Ville-Veikko Telkki

**Affiliations:** NMR Research Unit, Faculty of Science, University of Oulu P.O.Box 3000 90014 Oulu Finland otto.mankinen@oulu.fi ville-veikko.telkki@oulu.fi

## Abstract

Molecular exchange between different physical or chemical environments occurs due to either diffusion or chemical transformation. Nuclear magnetic resonance (NMR) spectroscopy provides a means of understanding the molecular exchange in a noninvasive way and without tracers. Here, we introduce a novel two dimensional, single-scan ultrafast Laplace NMR (UF LNMR) method to monitor molecular exchange using transverse relaxation as a contrast. The UF *T*_2_–*T*_2_ relaxation exchange spectroscopy (REXSY) method shortens the experiment time by one to two orders of magnitude compared to its conventional counterpart. Contrary to the conventional EXSY, the exchanging sites are distinguished based on *T*_2_ relaxation times instead of chemical shifts, making the method especially useful for systems including physical exchange of molecules. Therefore, the UF REXSY method offers an efficient means for quantification of exchange processes in various fields such as cellular metabolism and ion transport in electrolytes. As a proof of principle, we studied a halogen-free orthoborate based ionic liquid system and followed molecular exchange between molecular aggregates and free molecules. The results are in good agreement with the conventional exchange studies. Due to the single-scan nature, the method potentially significantly facilitates the use of modern hyperpolarization techniques to boost the sensitivity by several orders of magnitude.

## Abstract 2

An ultrafast two-dimensional NMR method allows quantification of molecular exchange rates efficiently based on *T*_2_ relaxation contrast.

## Graphical abstract

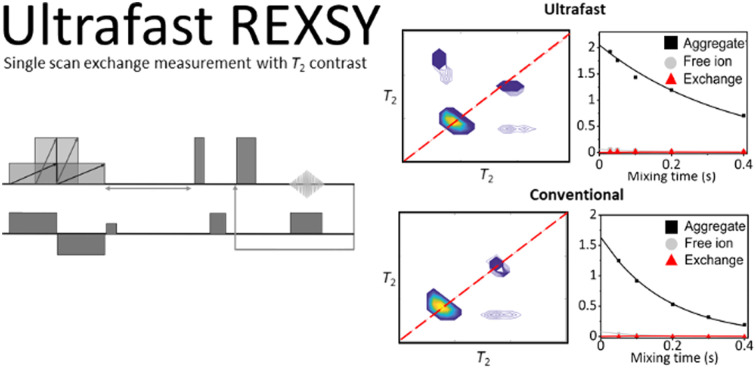

## 1. Introduction

Molecular exchange occurs due to diffusion or chemical transformations.^1,2^ When exchange occurs in a liquid or gas contained in a solid structure, its direct monitoring is challenging. Nuclear magnetic resonance spectroscopy (NMR) is used in the investigation of molecular motion in a noninvasive way, without tracers and it can be used in studying optically opaque samples.^3^ Different multidimensional NMR exchange methods, such as exchange spectroscopy (EXSY),^4^ diffusion exchange spectroscopy (DEXSY)^5,6^ and *T*_2_–*T*_2_ relaxation exchange spectroscopy (REXSY),^7–9^ have been exploited in the analysis of molecular exchange processes. For example, the REXSY method has been used to investigate exchange in urea-water systems,^7,10^ human cortical bone,^11^ geopolymers^12^ and shale,^13^ track pore-to-pore exchange,^14^ asphaltene adsorption,^15^ and water exchange in myelinated nerves^16^ and study aggregation of ionic liquids.^17^ These methods provide a two-dimensional map, in which the exchange sites and exchange are visible. However, these two-dimensional exchange NMR experiments must be repeated tens to hundreds of times with incremented evolution time or gradient strength, leading to long experiment times. Furthermore, the need for repetitions restricts the use of sensitivity increasing hyperpolarization methods.^18^

Ultrafast (UF) NMR spectroscopy, a concept introduced by Frydman *et al*,^19,20^ enables collection of multidimensional NMR data in a single scan. This technique is based on spatial encoding, where different evolution times are encoded into the different layers of the sample. After that, the encoded data are read using the principles of magnetic resonance imaging (MRI).^8,9^ As a result, the two-dimensional data is collected in a single scan and is analogous to the conventional, slow, counterpart.

Swisher *et al.*^21^ described a single scan UF EXSY method, which is based on small flip angle pulses, phase accrual during the spin echo times and collection of very few points in the indirect dimension. This method facilitates the collection of a series of data with variable mixing time just in a single scan. Therefore, it can be used in the full characterization of exchange process, although with reduced number of data points.

Spatial encoding has been exploited in *T*_1_ relaxation and diffusion experiments as well.^22–31^ Lately, we have introduced the spatial encoding technique to many multidimensional relaxation and diffusion experiments, which enable one to correlate various LNMR parameters such as *T*_1_, *T*_2_ and diffusion coefficient *D*.^30–36^ The inverse Laplace transformation (ILT) of the data provides relaxation time or diffusion coefficient distribution.^37^ Therefore, these methods are referred to as UF Laplace NMR (LNMR). UF LNMR has diverse application fields ranging from the identification of intracellular and extracellular metabolites^38^ to porous materials research,^36^ and the methods are applicable even with low field single-sided NMR instruments.^39–41^ The UF approach shortens the experiment time by one to three orders of magnitude.^42^ Due to the single-scan nature of UF LNMR methods, the use of hyperpolarization is feasible.^36^

Recently, we introduced the first UF LNMR exchange experiment, UF DEXSY, which relies on diffusion contrast.^35^ The experiment was exploited to study the exchange of water in a vesicle system formed by surfactant molecules. Here, we introduce a new ultrafast *T*_2_–*T*_2_ relaxation exchange spectroscopy (UF REXSY) method. We demonstrate its feasibility by quantifying molecular exchange of an ionic liquid (IL) system, which is a novel application area of UF LNMR. ILs are salts with melting point below 100 °C, and they are attractive for many applications such as organic synthesis, catalysis, lubrication, and electrochemistry due to their favorable properties such as low vapor pressure and flammability as well as high thermal stability.^43^

## 2. Experimental

### 2.1 Sample

The IL sample consisting of trihexyl(tetradecyl)phosphonium cations, [*P*_6,6,6,14_]^+^, and bis(mandelato)borate anions, [BMB]^−^ was previously prepared^17^ and reused in this study. Due to the previous ^129^Xe NMR analysis, the sample included also Xe gas. In the sample preparation, the residual moisture was removed from the IL in vacuum at 383 K for 4 hours. After that 300 μl of the IL was transferred in a 5 mm NMR tube and ^129^Xe gas at 1 atm was added to the NMR sample tube. Finally, the NMR tube was sealed by a flame.

### 2.2 Conventional REXSY experiments

**Fig. 1:**
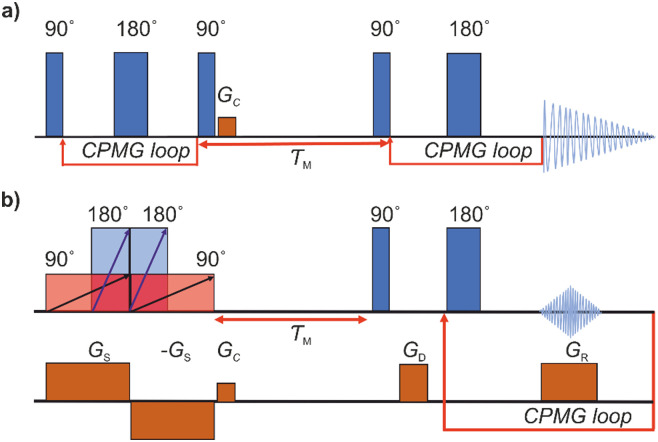
(a) Conventional and (b) ultrafast REXSY pulse sequences. Here, *τ*_M_ stands for the mixing period, and *G*_S_, *G*_C_, *G*_D_, and *G*_R_ refer to spatial encoding, crusher, dephasing, and read gradients, respectively.

As a reference data, we used *T*_2_–*T*_2_ data measured previously.^17^ To make results better comparable, the original data was cut to correspond roughly same echo time window as in the UF experiments. The conventional REXSY pulse sequence includes two CPMG blocks separated by a mixing time (Fig. 1a).^7^ Five experiments were performed at different mixing time varying from 50 to 400 ms. The relaxation delay was 3 s and echo time was 2 ms. After the first CPMG block, 1 ms crusher gradient *G*_c_ was applied to eliminate residual transverse magnetization. A total of 32 points was collected in each dimension with 8 scans and it took 8 hours to complete one experiment.

### 2.3 Ultrafast REXSY exchange experiments

The UF REXSY exchange experiments (Fig. 1b) were performed on a Bruker Avance III 400 MHz spectrometer at room temperature. The lengths of the 90° and 180° chirp pulses were 25 and 12.5 ms, respectively, and the sweep width Δ*ν* was 100 kHz. The strength of the spatial encoding gradient *G*_s_ was 133 mT m^−1^. The number of echoes in the CPMG loop was 32, the echo time was 5 ms, and each echo was consisted of 128 complex points. The strength of the trapezoidal read gradient *G*_R_ was 75 mT m^−1^ with the gradient ramp and stabilization times of 200 and 50 μs, respectively. Five experiments were performed varying the mixing time from 30 to 400 ms. The experiment time with 512 scans and the relaxation delay of 7 s was 1 hour. The Iterative Thresholding Algorithm for Multiexponential Decay (ITAMeD)^44^ was used for ILT analysis. For each map, 10 000 iterations, grid size of 25 by 25 and inversion limits from 0.001 to 1 s were used. The regularization parameter was chosen so that the inversion was stable and same value was used for all the maps for ensure comparison between measurements. The selection of suitable regularization parameter depends on the data quality and used ILT parameters such as grid size and inversion limits.

## 3. Results and discussion

The pulse sequence of the conventional REXSY experiment (Fig. 1a) has two CPMG blocks separated by the mixing period *τ*_M_.^7^ This experiment shows a relationship between the initial and final *T*_2_ relaxation time. The change in the *T*_2_ relaxation time indicates that physical or chemical environment of molecule changes during the mixing period. Conventional multidimensional NMR experiments, such as the REXSY experiment, are very time consuming, because data points of indirect dimension are collected individually in separate experiments. In fact, quite often the data of the direct dimension of the conventional REXSY experiment are collected point-by-point as well, leading to even much longer experiments. On the other hand, it is possible to accelerate the conventional REXSY by collecting all the echoes of the direct dimension in a single scan.

The pulse sequence for the UF REXSY experiment is shown in Fig. 1b. The sequence is identical to our previously published UF DEXSY sequence,^35^ but, as we describe below, it can be used also for UF REXSY measurements, when the gradient strengths are small enough and/or diffusion is slow.

The initial part of the sequence used for the single-scan spatial encoding of *T*_2_ of the indirect dimension consists of four adiabatic frequency-swept chirp pulses accompanied by two gradient pulses. The frequency of the chirp pulses increases linearly with time. On the other hand, the gradient pulses make the Larmor frequencies of the nuclei linearly dependent on position. Therefore, the first 90° chirp pulse excites the spins at the bottom layer first and the spins at the top layer last. The first 180° chirp pulse starts in the middle of the initial 90° pulse and both pulses switch off at the same time. Therefore, the length of 180° pulse is half of the length of 90° pulse. Technically, the overlapping 90° and 180° pulses were generated by the summation of two individual frequency-swept chirp pulses in the pulse shape tool of the spectrometer, and only single channel was needed for running the experiment. In principle, due to the initial 90° and 180° chirp pulses, a spin echo appears at the end of the pulses, and the echo time is linearly dependent on position, being zero at the top and equal to the length of the 90° pulse at the bottom. In practice, no echo is observed because spins in different layers experience different phases of the frequency-swept pulses.^45^ However, there is another pair of chirp and gradient right after the initial pulses to compensate the quadratic spatial dependence of the spin phases. In the second pair, the 180° chirp pulse overlaps with the first half of the 90° chirp pulse, and the gradient has an opposite polarity. Overall, the four chirp pulses accompanied by the bipolar gradient pulses form a double spin echo, and the double spin echo time *t*_1_ is linearly dependent on position *z*:^35^1
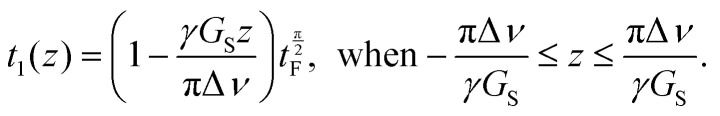
Here, *γ* is the gyromagnetic ratio, *G*_s_ is the amplitude of the spatial encoding gradient, Δ*ν* is the sweep width of the chirp pulses, and 
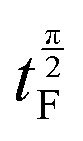
 is the length of the 90° chirp pulse. The double spin-echo amplitude is^35^2
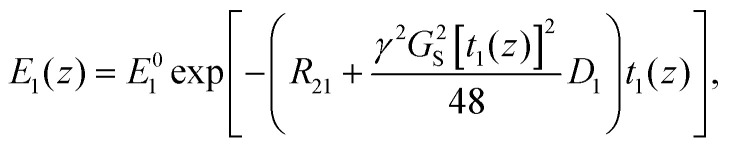
where *E*^0^_1_ is the initial signal amplitude, *R*_21_ is the transverse relaxation rate during the first *T*_2_ encoding, and *D*_1_ is the diffusion coefficient. The first term represents signal decay due to *T*_2_ relaxation and the second term due to diffusion.

The final part of the sequence (after the mixing time *τ*_M_) comprises of a CPMG loop for the second *T*_2_ encoding and reading data. The CPMG loop includes also gradients *G*_D_ and *G*_S_ for reading the spatial encoding information using the principles of 1D MRI.^46^ The echo amplitude in the CPMG loop in the second *T*_2_ encoding block is^35^3
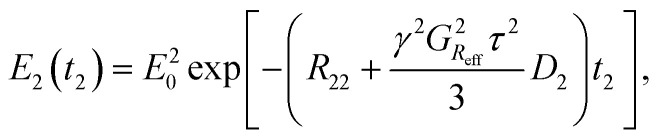
where *R*_22_ is the transverse relaxation rate during the second *T*_2_ encoding, *G*_Reff_ is the amplitude of the gradient pulse with the length of *τ* and the area corresponds to the area of read gradient *G*_R_, *τ* is the time between 90° and 180° pulses, *D*_2_ is the diffusion coefficient, and *t*_2_ is the variable in the second dimension. The first term in the parenthesis reflects the *T*_2_ decay, and the second term represents the diffusion decay.

If the sample includes several *T*_2_ components and if the diffusion terms are negligible, then, according to eqn (2) and (3), the overall signal observed in the UF REXSY experiment is4
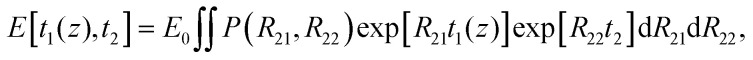
where *P*(*R*_21_,*R*_22_) is the 2D distribution of the relaxation rates. Therefore, UF REXSY enables one to collect data equivalent to the conventional REXSY experiment in a single scan. The relaxation time distribution can be resolved by a 2D ILT of experimentally observed UF REXSY data.^37^

**Fig. 2:**
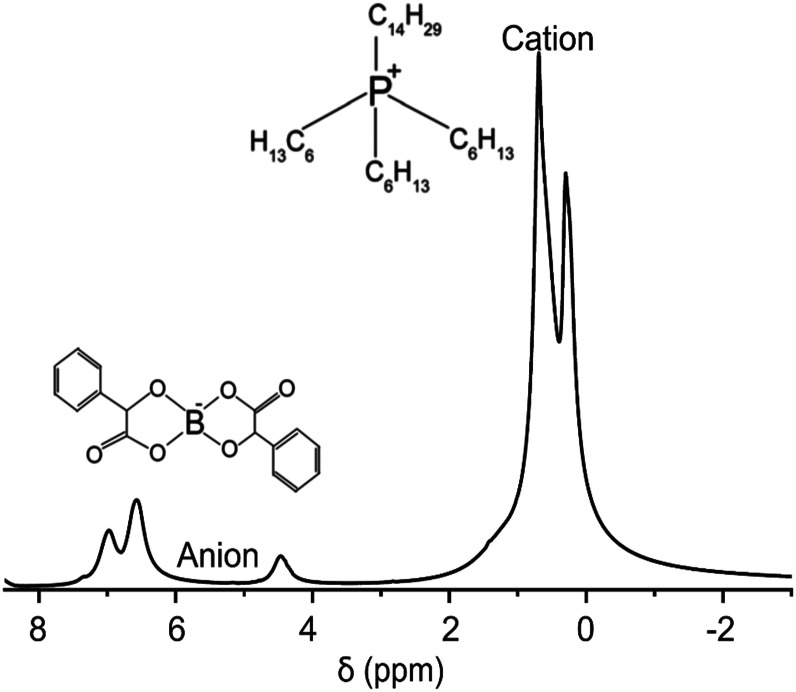
^1^H spectrum of the ionic liquid composed of trihexyl(tetradecyl)phosphonium, [*P*_6,6,6,14_]^+^, cation and bis(mandelato)borate, [BMB]^−^, anion measured at room temperature.

We demonstrate the feasibility of UF REXSY by studying a halogen-free orthoborate based ionic liquid consisting of the trihexyl(tetradecyl)phosphonium, [*P*_6,6,6,14_], cations, and bis(mandelato)borate, [BMB], anions (Fig. 2).^26^ In earlier studies, two significantly different diffusion components were observed from the IL.^47^ The smaller diffusion coefficient was proposed to arise from aggregates of ions and the higher from free ions. Our previous studies confirmed the existence of the two diffusion coefficients.^17^ With 2D *D-T*_2_ correlation experiments we were able to identify that the two diffusion coefficients are associated with two distinct *T*_2_ relaxation times. Our advanced relaxation modelling indicated that the aggregates include 10–70 ion pairs (cations and anions). Conventional REXSY revealed relatively slow exchange between the free ion and aggregate sites. The diffusion coefficients of both free ions and aggregates were observed to be very low, about 5 × 10^−12^ and 1 × 10^−13^ m^2^ s^−1^ at room temperature, respectively. ^1^H *T*_2_ values of free ions and aggregates are about 80 and 6 ms, respectively, both for cation and anion signals around 0–1 and 4–8 ppm (Fig. 2). Consequently, the IL is an excellent model system for proof-of-principles UF REXSY experiments.

**Fig. 3:**
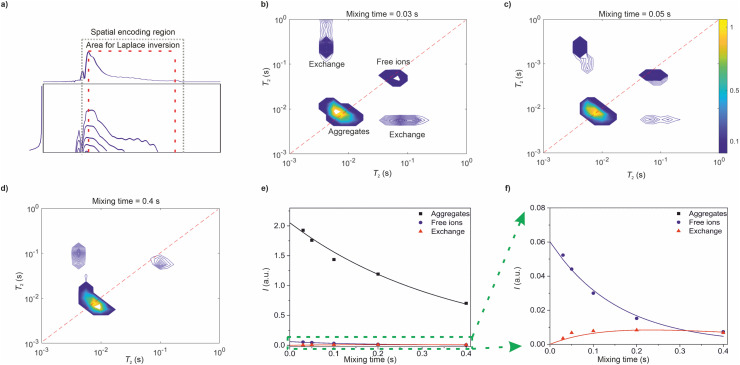
Ultrafast REXSY analysis of the ionic liquid sample at room temperature. (a) Raw data after Fourier transform along the spatial encoding direction measured with mixing time of 30 ms. The gray dotted lines indicate the spatial encoding region and the red dashed lines show the area selected for inverse Laplace transform. The first row and the first column, which was selected for Laplace inversion, are shown on top and left, respectively. (b–d) *T*_2_–*T*_2_ exchange maps resulting from 2D inverse Laplace transform of the raw data measured with mixing times of (b) 30, (c) 50 and (d) 400 ms. (e) Integrals of the *T*_2_–*T*_2_ peaks as a function of mixing time. Square, circle and triangle correspond to integrals of the aggregate, free ion and exchange peaks, respectively. Solid lines represent a fit of a two-site exchange model. (f) Zoom-in to the smaller integrals.

The raw data of the UF REXSY experiment after the Fourier transform along the spatial encoding direction is shown in Fig. 3a. The data was measured with the mixing time *τ*_M_ = 30 ms. The signal decays exponentially in both dimensions. Due to the small diffusion coefficients of the ions and relatively low gradient values used in the experiment, the signal decay is dominated by *T*_2_ relaxation, and the diffusion terms in eqn (2) and (3) are negligible. There are some artefacts at the edges of the spatial encoding region (indicated by gray dotted lines) due to imperfect performance of the chirp pulse in the beginning and at the end of the sweep.^48^ Furthermore, signal is slightly oscillating in the spatial encoding direction, most likely due to interference between the simultaneous 90° and 180° pulses. The data outside the spatial encoding region as well as the artificial regions close to the edges were removed.^48^

UF REXSY maps resulting from 2D inverse Laplace transform of the raw data are shown in Fig. 3b–d. The maps include two diagonal peaks around *T*_2_ = 8 and 80 ms corresponding to free ions and aggregates, respectively.^17^ Furthermore, there are off-diagonal cross-peaks revealing exchange between the aggregate and free ion sites. The exchange is physical, *i.e.*, the IL molecules are exchanging between the aggregate and free ion sites without chemical modifications in their molecular structures. The integrals of the signals as a function of mixing time are shown in Fig. 3e and f. The integral of the cross-peaks increases first with mixing time because increased number of exchanging ions. For cross-peak integral, average value of the two cross-peaks was used in the analysis. In some maps one of the exchange peaks is not visible due to their low intensity in general compared to the diagonal peaks. However, the loss of the less intense signal does not affect significantly to the result. At the longest mixing times it starts to decay due to *T*_1_ relaxation.

Molecular exchange rates were determined by fitting a two-site exchange model (eqn (22)–(25) in ref. 4) with the integral values of diagonal and off-diagonal peaks (solid lines in Fig. 3e and f). The resulting values are *k* = 1.7 ± 1.1 s^−1^, *k*_AI_ = 0.04 ± 0.03 s^−1^ and *k*_IA_ = 1.7 ± 1.1 s^−1^, where subscripts A and I refer to aggregates and ions, respectively. They are in good agreement with the values determined by the conventional reference measurements using roughly same echo time window (*k* = 1.8 ± 0.9 s^−1^, *k*_AI_ = 0.08 ± 0.04 s^−1^ and *k*_IA_ = 1.8 ± 0.9 s^−1^).

The UF REXSY experiment took about 1 h with 512 scans and 7 s relaxation delay. In contrast, the conventional REXSY experiment with 8 scans, 32 echoes in each dimension and 3 s relaxation delay took about 8 h. In the conventional experiment, both the direct and indirect dimensions were collected point-by-point, and reduced relaxation delay was used for shortening the extremely long experiment time. Theoretically, a single-scan UF REXSY experiment is 1024 times faster than a single-scan conventional experiment, if 32 time points are collected in each direction. On the other hand, it is possible to collect the direct dimension in a single scan also in the conventional experiment, reducing the UF acceleration to the factor of 32. The number of points in the UF experiments can be increased without time penalty. However, the spatial encoding decreases slightly the sensitivity of the experiment (typically by the factor of four),^36^ which may require higher number of scans collected in the UF experiment, if the sensitivity is a limiting factor. On the other hand, the single-scan UF approach significantly facilitates the use of hyperpolarization to boost the sensitivity of the experiment by several orders of magnitude.^18^

As explained earlier, the UF sequence shown in Fig. 1b can be used for both REXSY and DEXSY experiments. The type of the contrast depends on whether the signal decay is dominated by *T*_2_ relaxation or diffusion. As shown by eqn (2) and (3), the contrast can be adjusted by gradient strengths. Low gradient strengths result in *T*_2_ contrast while high values provide diffusion contrast. On the other hand, spatial encoding requires high enough gradient strengths. Therefore, the UF REXSY experiment is best suitable for samples with relatively low diffusion coefficients. In the case of faster diffusion, it is better to rely on diffusion contrast. In the case of more complex samples, the hard 90° and 180° pulses can be replaced by corresponding frequency selective pulses in the CPMG loop to select only desired signals from the spectrum.

## 4. Conclusions

In this article, we demonstrated the feasibility of a novel UF *T*_2_–*T*_2_ relaxation exchange spectroscopy (REXSY) NMR experiment and showed that it can be used for reliable quantification of exchange rates between aggregate and free ion sites in an ionic liquid sample, which is a new, interesting application field of UF LNMR. Depending on experimental parameters (*i.e.*, gradient strength) and diffusion coefficients, the same experiment can be used for *T*_2_ or diffusion contrast-based exchange experiments. The method significantly shortens the experiment time as compared to the conventional REXSY experiment, which is beneficial, *e.g.*, in the studies of samples that evolve or degrade over time. The method allows also improving resolution of the experiment in time efficient manner. Furthermore, the single scan approach facilitates the use modern hyperpolarization methods to boost sensitivity by several orders of magnitude, allowing low concentration studies even in physiological concentrations, and the observation window can be extended by using heteronuclei such as ^13^C with significantly longer *T*_1_ relaxation time than ^1^H.^36,38^ The method can be exploited to study physical or chemical exchange phenomena in many disciplines ranging from chemistry and biochemistry materials science, including protein-ligand binding, rearrangement of secondary structures of RNA, metabolic processes and exchange phenomena related to novel biomarkers.^38,49–51^

## Author contributions

M. S. U and O. M contributed equally to this work; M. S. U.: formal analysis, investigation, writing – original draft, visualization; O. M.: conceptualization, methodology, formal analysis, investigation, data curation, supervision; V. V. Z: conceptualization, data curation, funding acquisition, supervision; V.-V. T.: conceptualization, supervision, funding acquisition, project administration, writing – review and editing.

## Conflicts of interest

There are no conflicts to declare.

## Supplementary Material

CP-024-D2CP02944H-s001

## References

[cit1] Callaghan P. T., Coy A., MacGowan D., Packer K. J., Zelaya F. O. (1991). Nature.

[cit2] Inomata K., Ohno A., Tochio H., Isogai S., Tenno T., Nakase I., Takeuchi T., Futaki S., Ito Y., Hiroaki H., Shirakawa M. (2009). Nature.

[cit3] Callaghan P. T. , Translational dynamics and magnetic resonance: principles of pulsed gradient spin echo NMR, Oxford University Press, 2011

[cit4] Jeener J., Meier B. H., Bachmann P., Ernst R. R. (1979). J. Chem. Phys..

[cit5] Qiao Y., Galvosas P., Adalsteinsson T., Schönhoff M., Callaghan P. T. (2005). J. Chem. Phys..

[cit6] Callaghan P. T., Furó I. (2004). J. Chem. Phys..

[cit7] Lee J. H., Labadie C., Springer C. S., Harbison G. S., Lee J. H., Labadie C., Springer C. S., Harbison G. S. (1993). J. Am. Chem. Soc..

[cit8] Anderssen K. E., McCarney E. R. (2022). Food Control.

[cit9] D’Eurydice M. N., Montrazi E. T., Fortulan C. A., Bonagamba T. J. (2016). J. Chem. Phys..

[cit10] Dortch R. D., Horch R. A., Does M. D. (2009). J. Chem. Phys..

[cit11] Horch R. A., Nyman J. S., Gochberg D. F., Dortch R. D., Does M. D. (2010). Magn. Reson. Med..

[cit12] Li J., Mailhiot S., Sreenivasan H., Kantola A. M., Illikainen M., Adesanya E., Kriskova L., Telkki V. V., Kinnunen P. (2021). Cem. Concr. Res..

[cit13] Javed M. A., Komulainen S., Daigle H., Zhang B., Vaara J., Zhou B., Telkki V. V. (2019). Microporous Mesoporous Mater..

[cit14] Washburn K. E., Callaghan P. T. (2006). Phys. Rev. Lett..

[cit15] Shikhov I., Li R., Arns C. H. (2018). Fuel.

[cit16] Horch R. A., Gore J. C., Does M. D. (2011). Magn. Reson. Med..

[cit17] Javed M. A., Ahola S., Håkansson P., Mankinen O., Aslam M. K., Filippov A., Shah F. U., Glavatskih S., Antzutkin O. N., Telkki V. V. (2017). Chem. Commun..

[cit18] Telkki V. V. (2018). Magn. Reson. Chem..

[cit19] Frydman L., Scherf T., Lupulescu A. (2002). Proc. Natl. Acad. Sci. U. S. A..

[cit20] Pelupessy P. (2003). J. Am. Chem. Soc..

[cit21] Leon Swisher C., Koelsch B., Sukumar S., Sriram R., Santos R. D., Wang Z. J., Kurhanewicz J., Vigneron D., Larson P. (2015). J. Magn. Reson..

[cit22] McVeigh E., Yang A., Zerhouni E. (1990). Med. Phys..

[cit23] Pipe J. G. (1992). J. Magn. Reson..

[cit24] Thrippleton M. J., Loening N. M., Keeler J. (2003). Magn. Reson. Chem..

[cit25] Smith P. E. S., Donovan K. J., Szekely O., Baias M., Frydman L. (2013). ChemPhysChem.

[cit26] Shrot Y., Frydman L. (2008). J. Magn. Reson..

[cit27] Guduff L., Kurzbach D., van Heijenoort C., Abergel D., Dumez J. N. (2017). Chem. – Eur. J..

[cit28] Guduff L., Kuprov I., Van Heijenoort C., Dumez J. N. (2017). Chem. Commun..

[cit29] Ahola S., Mankinen O., Telkki V. V. (2017). Magn. Reson. Chem..

[cit30] Zhivonitko V. V., Ullah M. S., Telkki V. V. (2019). J. Magn. Reson..

[cit31] Urbańczyk M., Kharbanda Y., Mankinen O., Telkki V. V. (2020). Anal. Chem..

[cit32] Ahola S., Telkki V.-V. (2014). ChemPhysChem.

[cit33] Mankinen O., Hollenbach J., Ahola S., Matysik J., Telkki V. V. (2018). Microporous Mesoporous Mater..

[cit34] Tickner B. J., Zhivonitko V. V., Telkki V. V. (2021). Phys. Chem. Chem. Phys..

[cit35] Mankinen O., Zhivonitko V. V., Selent A., Mailhiot S., Komulainen S., Prisle N. L., Ahola S., Telkki V. V. (2020). Nat. Commun..

[cit36] Ahola S., Zhivonitko V. V., Mankinen O., Zhang G., Kantola A. M., Chen H. Y., Hilty C., Koptyug I. V., Telkki V. V. (2015). Nat. Commun..

[cit37] Song Y. Q., Venkataramanan L., Hürlimann M. D., Flaum M., Frulla P., Straley C. (2002). J. Magn. Reson..

[cit38] Zhang G., Ahola S., Lerche M. H., Telkki V. V., Hilty C. (2018). Anal. Chem..

[cit39] King J. N., Lee V. J., Ahola S., Telkki V.-V., Meldrum T. (2016). Angew. Chem., Int. Ed..

[cit40] Urbańczyk M., Kharbanda Y., Mankinen O., Telkki V. V. (2020). Anal. Chem..

[cit41] Kharbanda Y., Urbańczyk M., Zhivonitko V. V., Mailhiot S., Kettunen M. I., Telkki V. V. (2022). Angew. Chem., Int. Ed..

[cit42] Telkki V. V., Zhivonitko V. V. (2019). Annu. Rep. NMR Spectrosc..

[cit43] Freemantle M. , An introduction to ionic liquids, Royal Society of Chemistry, 2009

[cit44] Urbańczyk M., Bernin D., Koźmiński W., Kazimierczuk K. (2013). Anal. Chem..

[cit45] Tal A., Frydman L. (2010). Prog. Nucl. Magn. Reson. Spectrosc..

[cit46] Brown R. W. , Cheng Y. C. N., Haacke E. M., Thompson M. R. and Venkatesan R., Magnetic Resonance Imaging: Physical Principles and Sequence Design: Second Edition, John Wiley & Sons Ltd, Chichester, UK, 2014

[cit47] Filippov A., Shah F. U., Taher M., Glavatskih S., Antzutkin O. N. (2013). Phys. Chem. Chem. Phys..

[cit48] Telkki V. V., Urbańczyk M., Zhivonitko V. (2021). Prog. Nucl. Magn. Reson. Spectrosc..

[cit49] Ullah M. S., Zhivonitko V. V., Samoylenko A., Zhyvolozhnyi A., Viitala S., Kankaanpää S., Komulainen S., Schröder L., Vainio S. J., Telkki V. V. (2021). Chem. Sci..

[cit50] Baronti L., Guzzetti I., Ebrahimi P., Friebe Sandoz S., Steiner E., Schlagnitweit J., Fromm B., Silva L., Fontana C., Chen A. A., Petzold K. (2020). Nature.

[cit51] Qi C., Wang Y., Hilty C. (2021). Angew. Chem., Int. Ed..

