# Supplementary material for: Ultrafast transverse relaxation exchange NMR spectroscopy
Source: Phys Chem Chem Phys. 2022 Sep 2;24(36):22109–14. doi: 10.1039/d2cp02944h (PMC9491048; doi:10.1039/d2cp02944h)
Supplement: CP-024-D2CP02944H-s001 [file CP-024-D2CP02944H-s001.pdf]

Electronic Supplementary Material (ESI) for Physical Chemistry Chemical Physics.
This journal is © the Owner Societies 2022

Electronic supplementary information (ESI) for

Ultrafast transverse relaxation exchange NMR spectroscopy

Md Sharif Ullah, [a‡] Otto Mankinen, [a‡*] Vladimir V. Zhivonitko [a] and Ville-Veikko Telkki [a*]

[a] NMR Research Unit, Faculty of Science, University of Oulu, P.O.Box 3000, 90014 Oulu, Finland

* Corresponding authors

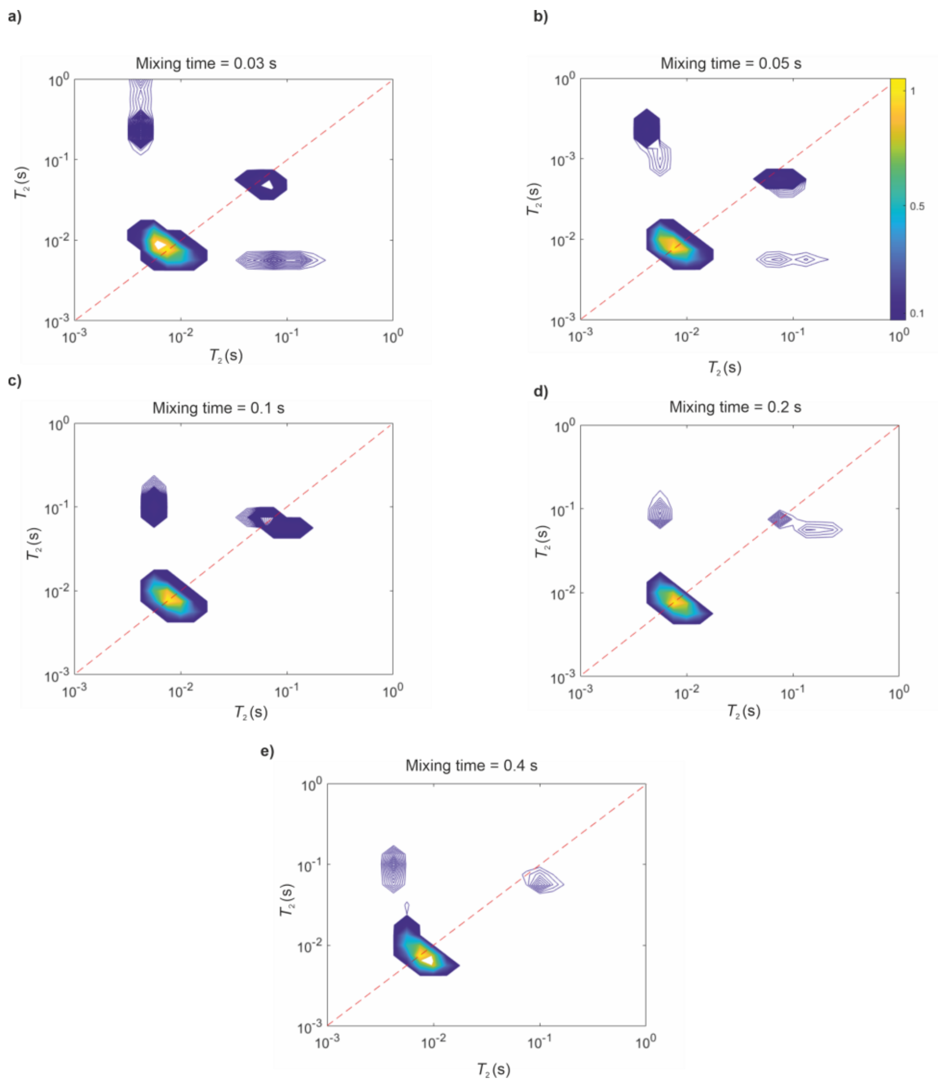

Fig S1. UF T_2-T_2 exchange maps resulting from 2D inverse Laplace transform of the raw data measured with mixing times of (a) 30, (b) 50, (c) 100, (d) 200 and (d) 400 ms.

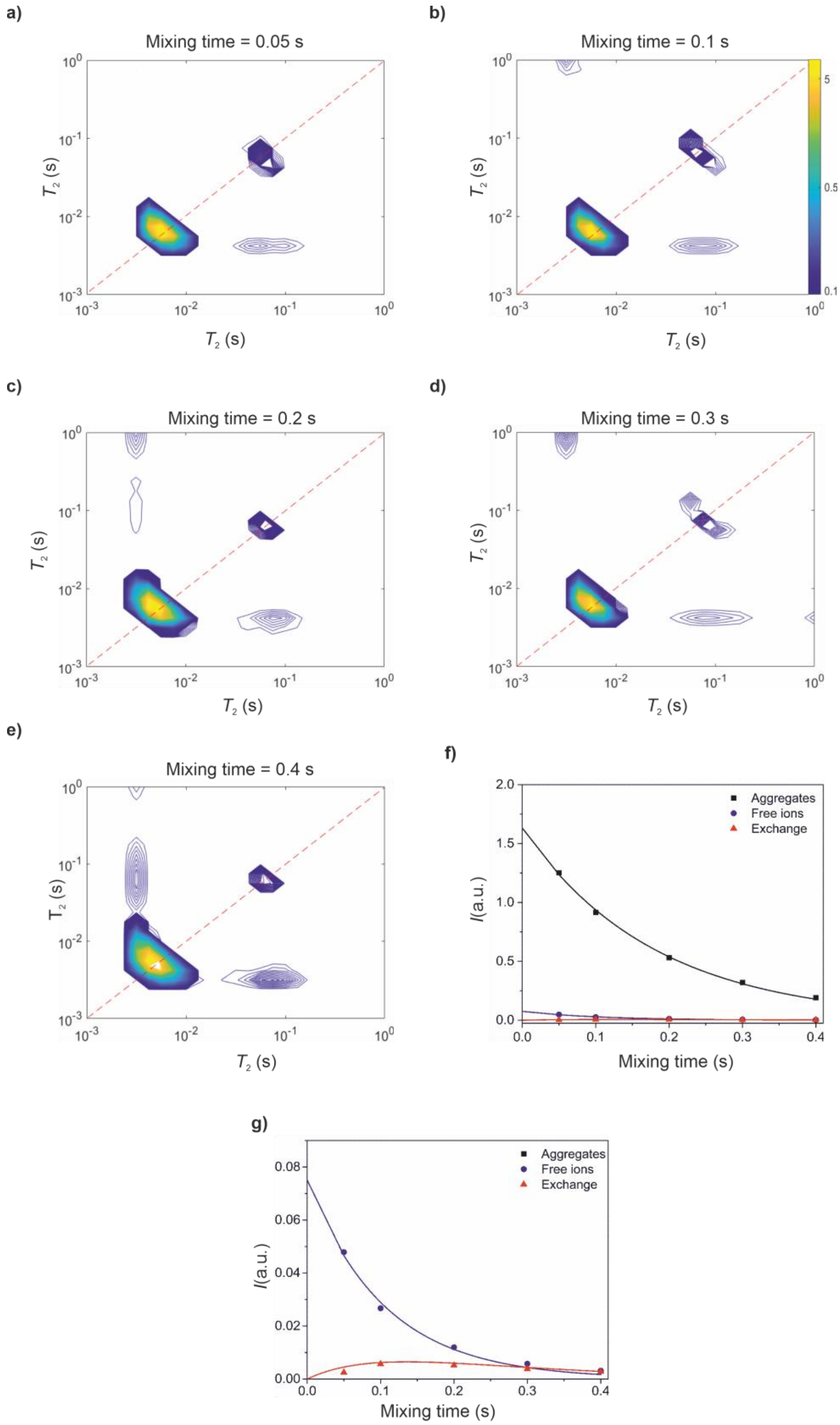

Fig S2. Conventional T_2-T_2 analysis of the ionic liquid sample at room temperature. (a-e) Conventional T_2-T_2 exchange maps resulting from 2D inverse Laplace transform of the raw data measured with mixing times of (a) 50, (b) 100, (c) 200 (d) 300 and (e) 400 ms. (e) Integrals of the T_2-T_2 peaks as a function of mixing time. Square, circle and triangle correspond to integrals of aggregate, free ion and exchange peaks, respectively. Solid lines represent a fit of a two-site exchange model. (g) Zoom-in to the smaller integrals.
